# Chicago Veterinary Society

**Published:** 1897-04

**Authors:** L. Campbell

**Affiliations:** Secretary


Secretary.

### CHICAGO VETERINARY SOCIETY.

The regular meeting was called to order March 11, 1897, by the President, Dr. Walker. The minutes of the previous meeting were read and approved.

Some remarks were made by the President in regard to veterinarians having the privilege from the Chicago Telephone Company to use the drug-store slot-telephones to call up their home-telephones free of charge. He requested that this matter be brought up under "new business."

A letter from Dr. Babb, Secretary of the Illinois State Veterinary Medical Association, was then read by the Secretary, setting forth the condition of the veterinary bill at Springfield, Ill. The report seemed favorable to the passage of the bill.

The Treasurer reported $37.01 in the treasury.

No report from the Secretary. No committees to report.

Under admission of new members, the applications of two gentlemen were considered—*i. e.*, Drs. E. W. McGarth and James Donovan. None of the Board of Censors being present to examine into the credentials of these two gentlemen, it was moved by Dr. Baker, seconded by Dr. McEvers, that the rules be suspended, and that as both gentlemen were well known to most of the members, both as to their qualifications as well as to their characters, that they be unanimously elected to membership. Voted; carried.

The regular programme being in order, the President called on Dr. Baker for his paper on "Open Joint." The paper proved to be an excellent one —one upon which the Doctor evidently had given much thought. It was particularly instructive as well as interesting, owing to the line of treatment suggested, which has been very successful, combined with the skill of the doctor, during the past five years. The paper was discussed by Drs. Allen, McEvers, Henderson, Ryan, Robertson, and Campbell. On motion, the discussion was closed. Motion by Dr. Robertson, seconded by Dr. Foster, that a vote of thanks be tendered to Dr. Baker by the Society for his most excellent paper. Voted and carried unanimously.

Dr. Robertson made some interesting observations in regard to the treatment of diseases of the hoof by horseshoers, and showed plainly the difficulty in drawing the line where the horseshoer should desist and the veterinarian begin, and showing that it was an impossibility for a horseshoer to shoe horses without doing some slight surgery (cutting corns, cracks, etc.) upon the hoof.

Referring to the remarks by the President in regard to the slot-machine telephones, Dr. Baker stated that he favored a conference with the company to request them to permit veterinarians the free use of the slot-telephones in drugstores for the purpose of calling up our own homes or offices, and he made a motion that the President appoint a committee of two to confer with the telephone company and try to arrange the matter. Seconded by Dr. Foster. On request of the members, the President appointed himself and Dr. Baker a committee.

A poster was next shown the Society, advertising free clinics and free treatment of horses at the Chicago Veterinary College for the benefit of poor people. Exception had been taken by one or two members to this placard, who had requested that the matter be brought up for consideration by the Society. Dr. Baker stated that all colleges had free clinics for the benefit not only of the students, but also in a measure as an assistance to the poor as well as to the sick or disabled patient; that people who would take advantage of the offer were either too poor to pay or deadbeats, and considering this fact, and also the fact that all this doctoring was done by the students for practice, he thought it a good thing and not at all in opposition to the rules of the Society or any harm to its members. Motion by Dr. Campbell, seconded by Dr. Broderick, that the Society drop this matter and take no further notice of the posters or the free clinics at the Chicago Veterinary College.

Motion by Dr. Campbell, seconded by Dr. Henderson, to return to Dr. Bennett the dues paid to the Society, as owing to the change in his location ordered by the Bureau of Animal Industry he would be unable to attend our meetings, the change having been ordered after he had attended only one of our meetings. A reconsideration was offered by Dr. Baker, who thought this would be a bad precedent. On vote the motion was lost.

On motion, adjournment. L. CAMPBELL, D.V.S., Secretary.

